# SU-8 cantilever with integrated pyrolyzed glass-like carbon piezoresistor

**DOI:** 10.1038/s41378-022-00351-9

**Published:** 2022-02-10

**Authors:** Jongmoon Jang, Giulia Panusa, Giovanni Boero, Juergen Brugger

**Affiliations:** 1 Microsystems Laboratory, École Polytechnique Fédérale de Lausanne (EPFL), 1015 Lausanne, Switzerland; 2 Department of Functional Ceramics, Korea Institute of Materials Science (KIMS), 51508 Changwon, Republic of Korea; 3 Optics Laboratory, École Polytechnique Fédérale de Lausanne (EPFL), 1015 Lausanne, Switzerland

**Keywords:** Electrical and electronic engineering, Materials science

## Abstract

Glass-like carbon (GC) is a nongraphitizing material composed entirely of carbon atoms produced from selected organic polymer resins by controlled pyrolysis in an inert atmosphere. The GC properties are a combination of the properties of glass, ceramic, and graphite, including hardness, low density, low thermal conductivity, high chemical inertness, biocompatibility, high electrical conductivity, and microfabrication process compatibility. Despite these unique properties, the application of GC in mechanical sensors has not been explored thus far. Here, we investigate the electrical, structural, and chemical properties of GC thin films derived from epoxy-based negative photoresist SU-8 pyrolyzed from 700 to 900 °C. In addition, we fabricated microGC piezoresistors pyrolyzed at 700 and 900 °C and integrated them into nonpyrolyzed SU-8 cantilevers to create microelectromechanical systems (MEMS) mechanical sensors. The sensitivities of the GC sensor to strain, force, surface stress, and acceleration are characterized to demonstrate their potential and limits for electromechanical microdevices.

## Introduction

The piezoresistive effect is a change in electrical resistivity when a material experiences mechanical strain^1^. This effect provides a direct energy/signal conversion from the mechanical to the electrical domain, which is widely used in microelectromechanical systems (MEMS)-based sensors, including pressure sensors^2^, accelerometers^3^, force sensors^4^, tactile sensors^5^, and flow sensors^6^. The sensitivity of a resistor to mechanical strain, called the gauge factor (*GF*), is usually defined as:^1^

1 $${{GF}} = \frac{{{\Delta}R}}{{R_0\varepsilon }} = \frac{{{\Delta}\rho }}{{\rho _0\varepsilon }} + 1 + 2\upsilon$$

where Δ*R*/*R*_0_ and Δ*ρ*/*ρ*_0_ are the relative variations in the electrical resistance and electrical resistivity, respectively. *ε* is the applied mechanical strain, and *υ* is Poisson’s ratio. Metal strain gauges (e.g., made of aluminum, gold, or copper) have *GF* of ~2, depending mainly on the dimensional change of the cross-sectional area and length corresponding to the Poisson ratio^7^. For semiconductors, the *GF* is more than one order of magnitude higher than the *GF* in metals (e.g*.*, p-type Si has a *GF* of ~100)^7^. These large *GF*s are caused by the large change of the electrical resistivity (Δ*ρ*), which, in turn, is due to the variation of the carrier density and of the mobility induced by the deformation of the band structure^8,9^. In polymer nanocomposites consisting of conductive nanoparticles in a polymer matrix, *GF* is determined by the tunneling-percolation between nanoparticles and the high flexibility of the polymer^10–12^.

Carbon allotropes, including graphite^13–15^, carbon nanotubes (CNTs)^16–18^, amorphous carbon (a-C)^4,19^, graphene^20,21^, and nanofoams^7^, have already been studied as piezoresistive materials. The diverse mechanical and electrical properties of structural allotropes are due to the different sp, sp^2^, and sp^3^ hybridized bonds, thus enabling a variety of sensor applications. In particular, graphite has been applied in the form of nanosheets/platelets to constitute polymer composites^13,14^. In the work by Ren et al*.*^15^, a graphite resistor drawn by a pencil was investigated. CNTs are used mainly as conductive materials in polymer composites for stretchable devices^16,17^. Zhao and Bai presented a graphite nanoplatelet/CNT hybrid nanocomposite in a polymer matrix to implement highly sensitive piezoresistors^18^. a-C thin films deposited by sputtering have been applied in MEMS force sensors, in which the *GF* can be tuned by the ratio of sp^2^/sp^3^ varying the DC bias voltage during sputtering^4,19^. The *GF* of graphene has been evaluated by transferring it to a silicon nitride membrane and used as a pressure sensor^20^. Zhao et al. reported on the enhancement of the *GF* of graphene by controlling the tunneling gap between nanographene islands and the application of this tunneling gap for ultrasensitive strain sensors^21^. Recently, Kardas and Pitkänen reported on the *GF* of carbon foam and its hierarchical hybrid structure with CNT/nanofibers^7^.

Glass-like carbon (GC), also known as glassy carbon or vitreous carbon, is a disordered sp^2^-carbon allotrope classified as a nongraphitizing carbon that cannot be converted into crystalline graphite even at temperatures of 3000 °C^22,23^. GC combines the properties of glass, graphite, and ceramics^24^, which includes a high-temperature resistance, extreme chemical stability, hardness with low density, impermeability to gases and liquids, electrical conductivity, and biocompatibility with blood and tissues^25–28^. Due to its excellent material features, numerous studies based on GC have been reported, such as nanolattices with a high strength-to-density ratio^25,26,29^, electrodes for biomedical devices or batteries^30–33^, and gas-sensor platforms with nanomeshes/wires^34–36^. GC is obtained by controlled thermal degradation of a polymer precursor (e.g*.*, phenol-formaldehyde (PF) resins or polyfurfuryl alcohol)^37^ beyond its decomposition temperature in an inert atmosphere. Specifically, micro-/nanoscale devices in GC can be made via pyrolysis after photolithographically patterning photosensitive PF resins such as SU-8, polyimide, and AZ9260^38–40^. GC has already been investigated in the MEMS research field, e.g*.*, for electrodes and sensor platforms with micro-/nanostructures. It is thus of interest to evaluate whether GC exhibits any form of piezoresistivity so that GC can be considered a potential mechanical force or strain sensor, which would widen the use of GC structures for MEMS applications, in particular where biocompatibility, temperature resistance, and chemical inertness are required. In a paper from 1976, Hunt et al. investigated the resistivity change of a GC rod at the cm scale under tensile stress and experimentally confirmed that the piezoresistivity of GC follows an earlier theoretical model that describes GC as consisting of narrow curved and twisted ribbons^41^. Hunt et al*.* showed in particular that a higher pyrolysis temperature leads to a reduction in the piezoresistive effect in GC due to the suppression of energy states that exist at lower pyrolysis temperatures. In their paper, Hunt et al*.* also stated that “deformation at lower heat treatment would cause the conduction band edge to exclude a relatively larger number of available energy states than at high heat treatment. From this argument, one would expect the piezoresistance effect to be larger at lower heat treatment”, which is indeed an effect that we observe in our studies, as we will see later. After the work of Hunt et al*.*, no other studies on the piezoresistivity of GC have been reported thus far, except that our preliminary experiment is presented as a conference proceeding^42^. A recently proposed structural model of GC as a fullerene-like element composed of randomly distributed curved graphene fragments^22^ has stimulated scientific interest in phenomena that could derive from the interaction of the internal elements of GC caused by mechanical strain. In addition, materials similar to GC have been investigated for mechanical sensors. For example, Dai et al*.*^43^ and Wang et al*.*^44^ reported on the piezoresistive properties of glassy graphene and carbonized silk fabrics, respectively.

In this study, we focus on GC derived from SU-8, a polymer that is well known for its efficient patternability by lithography. Briefly, our approach first creates a GC element from an SU-8 thin film by pyrolysis at temperatures of 700 and 900 °C, which is subsequently integrated into a second nonpyrolyzed SU-8 thick layer that is lithographically shaped as a cantilever. Therefore, we propose a new hybrid SU-8 MEMS device that contains locally pyrolyzed SU-8 (i.e*.*, glass-like carbon) piezoresistive elements. In our systematic study, we first analyzed GC thin-film elements for their electrical properties (such as electrical resistivity, Hall mobility, and carrier concentration). Then, the material properties of the GC thin films were characterized by Raman spectroscopy and X-ray photoelectron spectroscopy (XPS), and their nanostructure was investigated by transmission electron microscopy (TEM). Furthermore, the *GF*s of the GC piezoresistors are obtained by measuring the relative resistance change when mechanical strain is applied to the SU-8 cantilever. Moreover, the force/surface stress sensitivity and the dynamic response of the GC-based MEMS sensor were investigated.

## Results and discussion

**Fig. 1:**
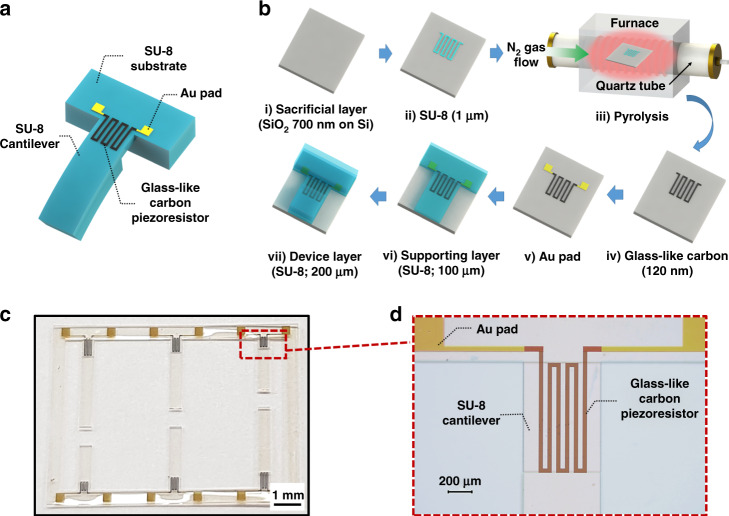
Design and fabrication of the glass-like carbon (GC)-based sensor. **a** Schematic drawing of the GC strain sensor, and **b** its fabrication process. **c** Optical image of six fabricated GC strain sensors with different lengths in the range from 2.5 to 5.0 mm. **d** Enlarged optical microscopic image of a fabricated GC piezoresistor

To assess the piezoresistive properties of GC thin films, we fabricated conducting GC gauges embedded into photolithographically patterned SU-8 cantilever structures, as shown in Fig. 1a. Due to its lower Young’s modulus and microfabrication versatility^45^, SU-8 has often been applied to MEMS devices such as accelerometers^11^, atomic force microscopy (AFM) cantilevers^46^, and acoustic sensors^47^ as an alternative to harder materials such as silicon. Figure 1b shows the fabrication process for the GC-based conducting element integrated into the SU-8 cantilever. A 700 nm-thick SiO_2_ layer is wet oxidized on the Si wafer and used later as a sacrificial layer to release the final device from the substrate (i). A 1 µm-thick SU-8 layer is patterned using negative photolithography (ii) to form the green body of the subsequent GC structures. The GC structures are obtained by pyrolysis of the SU-8 green body inside a nitrogen flow atmosphere at different pyrolysis temperatures (*T*_*p*_) for 1 h (iii). The realized GC structures, called GC700 and GC900, are manufactured at *T*_*p*_ of 700 and 900 °C, respectively (iv). During the pyrolysis process, most of the shrinkage is generated in the vertical direction and corresponds to an ~85–88% reduced thickness depending on the pyrolysis temperature. For the electrical connection of the GC structures, a 50 nm-thick Au film is deposited by sputtering and patterned by lift-off to form electrical leads (v). To integrate the GC structure as piezoresistive elements into the SU-8 cantilever, a 100-µm-thick SU-8 support layer is spin-coated over the GC element and Au wiring and lithographically patterned in alignment with the GC and Au structures (vi). To avoid cracks in SU-8 due to the residual stress induced by UV exposure and post baking, a low UV exposure intensity (350 mJ/cm^2^) and uncommonly long duration (12 h) at low temperature (45 °C) are used^47^. An additional 200 µm-thick SU-8 structure is patterned to realize the device layer used to frame and support the SU-8 cantilever after release. In one design variation, for the GC700 sample, an additional proof mass for the dynamic mode^48^ is formed with the device layer covering 80% of the cantilever length, excluding the part with the piezoresistive pattern (vii). The same UV exposure and post bake conditions to minimize the residual stress are also applied for the device layer and proof mass. Finally, the sacrificial layer is removed with a buffered oxide etchant (BOE) to create an array of free-standing SU-8 cantilevers with GC piezoresistors. Figure 1c shows the fabricated GC piezoresistor-based SU-8 MEMS sensor array of six cantilevers. The length of each cantilever varies between 2.5 and 5.0 mm, as shown in Table 2. The thickness and width of all fabricated SU-8 cantilevers are identical and are 100 and 700 µm, respectively. Figure 1d shows a microscopic image of the GC structure, which has a ‘meander shape’ similar to typical metallic strain gauges. The width of all GC resistors is 40 µm, and the length of the GC piezoresistive meander (*L*_*p*_) is ~20% of the length of the SU-8 cantilevers (*L*), as shown in Figure S1 in the Supplementary Information. As shown in Fig. 1c, d, there is no noticeable intrinsic bending or deformation of the freestanding cantilevers. GC and Au are successfully transferred to the SU-8 support.

**Fig. 2:**
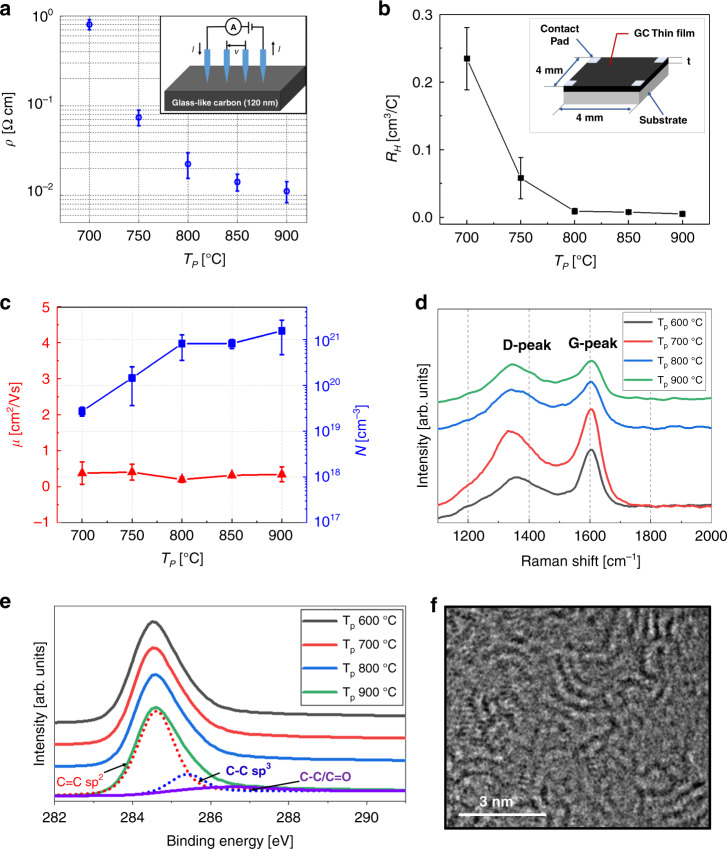
Glass-like carbon (GC) thin film characterization. **a** Resistivity *ρ* of the GC thin films, measured by a four-point probe system, as a function of the pyrolysis temperature *T*_*p*_. **b** Hall coefficient *R*_*H*_, and **c** carrier mobility *μ*_*H*_ and concentration *N* of GC thin films for *T*_*p*_ from 700 to 900 °C. **d** Raman spectroscopy, and **e** X-ray photoelectron spectroscopy (XPS) C1s spectra for the GC thin films obtained at a *T*_*p*_ of 600–900 °C. **f** Transmission electron microscopy images of the GC thin film obtained at a *T*_*p*_ of 900 °C

Prior to device characterization, the material properties were evaluated for GC thin films prepared by pyrolysis at 700– 900 °C for 1 h using a 1-μm-thick cured SU-8 photoresist as the precursor (green body) on the Si wafer. Considering that the resistance of the GC thin film is too high at 600 °C for electronic device applications and that the increase in GC conductivity is not significant at *T*_*p*_ above 900 °C^49^, we set the range of pyrolysis temperatures between 700 and 900 °C. For electrical characterization, the sheet resistance (*R*_*s*_) and thickness (*t*) of each GC thin film were measured (Table S1 in the Supplementary Information). As the pyrolysis temperature *T*_*p*_ increases, *R*_*s*_ significantly decreases, especially in the range from 700 to 800 °C. The thickness of the GC thin films formed by pyrolysis tends to be thinner at higher *T*_*p*_ with a vertical shrinkage of ~85–88%, and the thickness for each *T*_*p*_ is reported in Table S1 of the Supplementary Information. Figure 2a shows the resistivity (*ρ*) of the GC thin films as a function of *T*_*p*_. The measured resistivity of the GC thin films at a *T*_*p*_ of 900 °C is ~1.1 ± 0.26 × 10^−2^ Ω cm, i.e*.*, similar to the measured resistivity of highly doped p-type silicon^50^. In addition, the Hall effect was measured to estimate the density (*N*) and mobility (*μ*_*H*_) of the dominant charge carriers that contribute to the electrical conductivity (σ) of GC^50^. As shown in the inset of Fig. 2b, we used a GC thin film formed on a Si wafer having a 700-nm-thick SiO_2_ insulating layer with a size of 4 mm × 4 mm. The varying thickness of the GC thin film according to *T*_*p*_ is listed in the Supplementary Information (Table S1). Figure 2b shows the Hall coefficient (*R*_*H*_) of the GC thin films as a function of *T*_*p*_, and *R*_*H*_ decreases as *T*_*p*_ increases. The dominant charge carriers in our GC samples are holes for all values of *T*_*p*_, in agreement with previous studies in which the Fermi level lies inside the valence band due to localized states for *T*_*p*_ > 600 °C^51^. Figure 2c shows the carrier density (*N*) and Hall mobility (*μ*_*H*_) of GC thin films as a function of *T*_*p*_. The carrier density increases from 0.27 to 16 × 10^20^ cm^−3^ (see Table S2 in the Supplementary Information). The mobility is not significantly dependent on *T*_*p*_ and ranges from 0.20 to 0.41 cm^2^/(Vs), which is approximately one order of magnitude lower than the mobility of commercial bulk GC^52^, possibly due to the difference between bulk and thin-film properties or to the significantly higher pyrolysis temperature (1000–2800 °C) used to produce typical commercial GCs^53^. In addition to the electrical properties, the structural characteristics of the material of the GC thin films are evaluated by first-order Raman spectra, as shown in Fig. 2d. The two main peaks between 1200 and 1700 cm^−1^ are typically used to characterize graphitic carbons^28^. The peak at 1350 cm^−1^, called the disorder-induced (D-band) peak, is not observed in perfectly aligned single-crystal graphite. The peak at 1600 cm^−1^, called the graphitic (G-band) peak, is associated with the in-plane sp^2^-bonded carbon atoms. The integrated intensity ratio of the D-band (*I*_*D*_) to the G-band (*I*_*G*_) peaks is proportional to the in-plane size of the sp^2^ cluster (*L*_*a*_)^4,28^. For the SU-8-derived GC thin films in this study, the ratio *I*_*D*_*/I*_*G*_ shows a tendency to increase with *T*_*p*_, as shown in Table 1. Detailed fitted spectra are shown in the Supplementary Information (Fig. S2). This trend is also observed when *T*_*p*_ is increased to 900 °C and is similar to the trend previously reported for pyrolytic carbons created by other types of precursors, such as polyfurfuryl alcohol, cellulose, and wood^28^. For the chemical assessment of the sp^2^ and sp^3^ content of the GC thin films pyrolyzed at 600 to 900 °C, XPS C1s spectra and the detailed fitting are measured, as shown in Fig. 2e and Supplementary Information (Fig. S3), respectively. Typically, the sp^2^ (C=C), sp^3^ (C–C), and C–O/C=O hybridizations have binding peaks at 284.5, 285.3, and 286.4 eV, respectively^54^. The percentage of the sp^2^ contents of the GC thin films increases as a function of *T*_*p*_ from 74 to 86%, as shown in Table 1, indicating that the GC thin films consist mainly of sp^2^ constituting a graphite layer or cluster of fullerenes, and their fraction increases with *T*_*p*_. Figure 2f shows the nanostructure of the SU-8-derived GC at *T*_*p*_ = 900 °C, as observed by TEM, consisting of fullerene-like disordered carbon clusters. As *T*_*p*_ increased from 600 to 800 °C, the curled layers became longer and tended to form stacks (see Figure S4 in Supplementary Information). This tendency of the internal nanostructure to change with increasing *T*_*p*_ is similar to the tendency shown in previous studies. By further thermal treatment up to 2500 °C, the graphene-like curled layers become more elongated and gradually become well-organized onion-like fragments^28,55–57^. The surface morphology of the GC thin film was analyzed by scanning electron microscopy and AFM (see Figs. S5 and S6, respectively, in the Supplementary Information) according to the *T*_*p*_ in the range from 600 to 900 °C. The surface roughness (*R*_*a*_) of each GC thin film is less than 2.5 nm, and there are no significant differences among the samples pyrolyzed at different temperatures.

**Table 1:** The integrated intensity ratio of *I*_*D*_*/I*_*G*_ and sp^2^ contents of the SU-8-derived glass-like carbon thin film pyrolyzed for 1 h each at temperatures from 600 to 900 °C

Pyrolysis Temperature [°C]	600	700	800	900
*I*_*D*_*/I*_*G*_	2.06	2.10	2.15	2.16
sp^2^ contents [%]	74	76	82	86

**Fig. 3:**
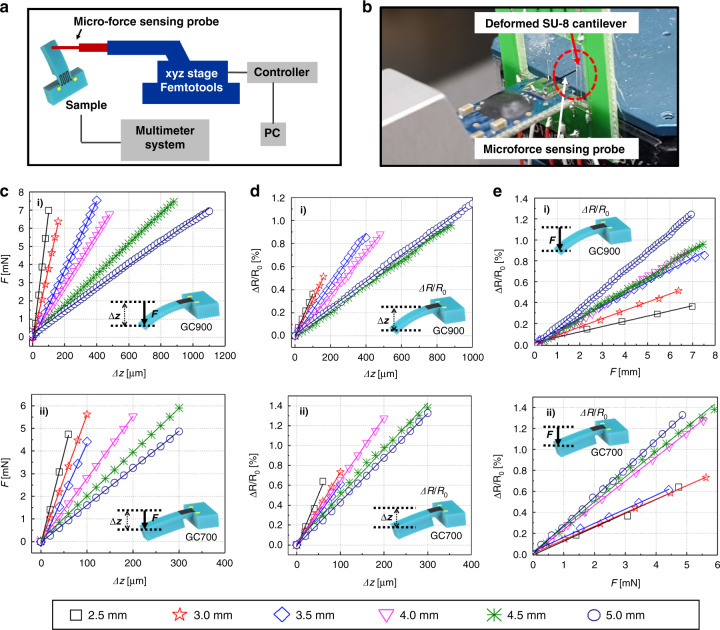
Static response of the glass-like carbon (GC)-based piezoresistive sensors. **a** Schematic diagram and **b** image of the measuring apparatus used to characterize the GC-based piezoresistive sensors. **c** Measurement of the bending force *F* as a function of the applied displacement *∆z*. **d** Relative resistance variation as a function of the applied displacement *∆z*. **e** Relative resistance variation as a function of the applied force *F*. Panels (i) and (ii) report the results of measurements performed on the GC900 and GC700 sensors, respectively. Different symbols indicate different cantilever lengths, and the corresponding lengths are shown at the bottom of the figure

For device characterization, a microforce sensing probe (FT-S1000, FemtoTools AG, Switzerland) with a precise xyz-axis manipulator is used to measure the spring constant of the fabricated SU-8 cantilevers, as illustrated in Fig. 3a. An optical image of the mechanically deflected SU-8 cantilever is shown in Fig. 3b. The spring constants *k* of the SU-8 cantilevers with a uniform cross-section along the length of the beam are calculated as:^58^

2 $$k = - \frac{F}{{{\Delta}z}} = \frac{{3\left( {EI} \right)_e}}{{l^3}},$$

where *F*, *l*, and Δ*z* describe the applied force, length, and deflection of the SU-8 cantilevers, respectively. *E* is Young’s modulus of SU-8, and *I* is the moment of inertia of the cantilever. *(EI)*_*e*_ denotes the effective bending stiffness of the SU-8 cantilever. Figure 3c (i) shows the measured force (*F*) as a function of the deflection of the GC900-based SU-8 cantilevers along the z-direction (Δ*z*). The spring constant *k*, which is obtained from the linear fit of the curve, increases as the length of the cantilever decreases, as shown in Table 2. The spring constant is also simulated by finite element analysis (FEA; see Fig. S7 in Supplementary Information), which is compared with the measured *k* in Table 2. The mechanical stiffness of the GC700-based SU-8 cantilevers is also characterized by measuring the bending force *F* as a function of the applied displacement along the z-direction Δ*z* (Fig. 3c(ii)). Due to the influence of the proof mass, the spring constants of GC700 are higher than the spring constants of the GC900-based cantilevers and are listed in Table 2. The FEA results are also shown in the Supplementary Information (Fig. S7). The relationship between the relative variation in the resistance (Δ*R*/*R*_0_) and the strain (*ε*) applied to the piezoresistive layer can be expressed as:^58,59^

3 $$\frac{{{\Delta}R}}{{R_0}} = {\rm{GF}}\varepsilon .$$

**Table 2:** Measured length, spring constant, resistance, gauge factor (*GF*), sensitivity to the input force of the glass-like carbon (GC)-based SU-8 sensors; GC900 and GC700 indicate the GC samples pyrolyzed at 900 and 700 °C, respectively

Length of the cantilevers [mm]	2.5	3.0	3.5	4.0	4.5	5.0
GC900 sample (w/o proof mass)						
Spring constant [N/m]	72	41	19	14	8.4	6.3
Spring constant [N/m] (Simulation)	50	29	18	12	8.4	6.1
R_0_ [MΩ]	0.14	0.16	0.18	0.20	0.23	0.26
Gauge factor	1.7	2.2	2.0	2.2	1.8	2.1
Force sensitivity (Δ*R*/*R*_0_*F*) [N^-1^]	0.5	0.8	1.2	1.3	1.3	1.8
GC700 sample (w/proof mass)						
Spring constant [N/m]	79	57	45	29	19	16
Spring constant [N/m] (Simulation)	86	50	31	21	15	11
R_0_ [MΩ]	20	22	28	31	35	38
Gauge factor	2.7	2.7	3.0	4.2	3.9	4.5
Force sensitivity (Δ*R*/*R*_0_*F*) [N^−1^]	1.3	1.3	1.4	2.3	2.4	2.7

The strain (*ε*) can be computed as:^58^

4 $$\varepsilon = \frac{{3\left( {1 - \frac{{L_p}}{{2L}}} \right)Z_{nr}{\Delta}z}}{{L^2}},$$

where *L* and *L*_*p*_ are the length of the SU-8 cantilever and the length of a GC piezoresistive ‘meander shape’ (*L*_*p*_), respectively. *Z*_*nr*_ is the distance of the GC-resistor layer from the neutral axis of the cantilever. Since the thickness of SU-8 is dominant with respect to the thickness of the GC (~1000 times thinner than SU-8), we can assume that the cantilever consists of SU-8 and an infinitely thin GC layer. Substituting Eq. (3) into Eq. (4), the *GF* can be written as:

5 $$\begin{array}{ll}{{GF}} = \frac{{{\Delta}R}}{{R_0}}\left( {\frac{{3\left( {1 - \frac{{L_p}}{{2L}}} \right)Z_{nr}{\Delta}z}}{{L^2}}} \right)^{ - 1}\\\qquad = \frac{{{\Delta}R}}{{R_0{\Delta}z}}\left( {\frac{{3\left( {1 - \frac{{L_p}}{{2L}}} \right)Z_{nr}}}{{L^2}}} \right)^{ - 1}.\end{array}$$

Figure 3d (i) shows the variation of the resistance Δ*R*/*R*_0_ as a function of the vertical deflection Δ*z* of the GC900-based strain sensor and for different lengths of the cantilevers. The term (Δ*R*/Δ_0_)(Δ*z*)^−1^ is obtained from the linear fit of the curves shown in Fig. 3d (i), and *GF* is calculated using Eq. (5). The average measured *GF* for the GC900-based strain sensor is 2.0. The measured *GF* for the SU-8 cantilevers with lengths between 2.5 mm and 5.0 mm are listed in Table 2. The *GF* of the characterized GC900 is much lower than the *GF* of semiconductor materials (~ 100) and similar to the *GF* of metals (~2)^1,9^. The comparison with other carbon allotropes is shown in Table 3. The measured *GF* of GC900 is similar to the *GF* of untuned single-layer graphene (*GF* ≈ 1.6)^20^. Let us now evaluate the GC700 device, i.e*.*, pyrolyzed at 700 °C instead of 900 °C. The variation in the resistance as a function of the mechanical displacement for the GC700-based cantilevers is shown in Fig. 3d (ii). Since the average strain applied to the GC piezoresistor cannot be obtained by Eq. (4), the average strain is simulated by FEA where the applied strain of GC700 is ~1.9 times higher than the applied strain of GC900 when the same deflection is applied (see Fig. S8 in Supplementary Information). The measured *GF* values considering the effect of the proof mass are reported in Table 2. For the GC700-based sensors, the average *GF* is 3.5, which is ~1.8 times larger than the average *GF* of the GC900 sensors. This result is consistent with the previous study by Hunt et al*.*^41^, in which the intrinsic piezoresistivity of GC gradually decreased as the *T*_*p*_ was increased from 600 to 1100 °C (the piezoresistive coefficient was zero at *T*_*p*_ ≥ 1100 °C). As in the case of metals, the piezoresistive properties of the GC sensors are attributed mainly to geometrical effects, but the GC700 sensor has intrinsic piezoresistivity, although modest compared to semiconducting materials. A previous study reported that the intrinsic piezoresistivity of GC is due to the band gap formed by the twisted ribbon network, which depends on *T*_*p*_ based on the early structural model of the GC^41^. In our study, we also observed intrinsic structural changes in GC, such as an increase in the in-plane size of the sp^2^ cluster (*L*_*a*_) and the alignment of fullerene-like layers as *T*_*p*_ increased, which probably affected the intrinsic piezoresistivity of the GC.

**Table 3:** Comparison of the performance of carbon-based piezoresistive sensors

Types of carbon allotrope	Fabrication process	Gauge factor	Force sensitivity
Graphite^15^	Drawing by pencil	N.A.	0.9 [mV/mN]
Amorphous Carbon (a-C)^19^	DC magnetron sputtering	6.9	0.01 [N^−1^]
Hydrogenated a-C (a-C:H)^66^	PACVD	40–90	N.A.
Graphene^20,21^	CVD^20^ RPECVD and Tunneling^21^	1.6^20^ 300^21^	8.5 [mV/bar]^20^ N.A.
Glass-like Carbon (In this work)	SU-8 precursor and Pyrolysis	2.0 (*T*_*p*_: 900 °C) 3.5 (*T*_*p*_: 700 °C)	1.2 [N^−1^] (*T*_*p*_: 900 °C) 1.9 [N^−1^] (*T*_*p*_: 700 °C)

The relative resistance variations Δ*R*/*R*_0_ of the GC900 and GC700 cantilevers as a function of the applied force *F* are shown in Fig. 3e (i) and (ii), respectively. As the length of the cantilever increases, the relative resistance variation for both strain sensors increases. The average force sensitivity *(∆R/R*_*0*_*F*) obtained from each cantilever length from 2.5 to 5 mm of the GC900 sensor is 1.2 N^−1^. For the GC700 sensor, the average force sensitivity from 2.5 to 5 mm of each cantilever is 1.9 N^−1^, which is higher than the average force sensitivity of the GC900 (see details in Table 2).

The surface stress on the GC-based SU-8 cantilever is characterized in view of its potential applications as sensors that detect surface stress, such as in AFM, biochemical, and gas sensors. The equation below has been used to compute the surface stress (*σ*_*S*_) induced in multilayer cantilevers:^58,59^

6 $$\frac{{{\Delta}R}}{{R_0}} = - GF\left( {\frac{1}{{\mathop {\sum }\nolimits_i E_ih_i}} + \frac{{Z_TZ_R}}{{\mathop {\sum }\nolimits_i E_ih_i\left( {Z_{in}^2 + \frac{1}{3}\left( {\frac{{h_i}}{2}} \right)^2} \right.}}} \right)\sigma _s,$$

where *Z*_*T*_ and *Z*_*R*_ describe the positions of the top layer and piezoresistive layer, respectively. *E*_*i*_ is the Young’s modulus of the i^th^ layer, and *h*_*i*_ and *Z*_*in*_ are the thickness and position of the i^th^ layer with respect to the neutral axis. Since the thickness of SU-8 is dominant with respect to the thickness of the GC, Eq. (6) can be approximated by:

7 $$\frac{{{\Delta}R}}{{R_0}} = - {{GF}}\left( {\frac{1}{{Eh}} + \frac{3}{{Eh}}} \right)\sigma _s = - {{GF}}\left(\frac{4}{{Eh}}\right)\sigma _s$$

where *E* and *h* are Young’s modulus and the height of the SU-8 cantilever, respectively. Therefore, the average electrical sensitivities to the surface stress can be computed as *GF(4/Eh)*, assuming *E* = 4 GPa^60^, which gives 0.02 and 0.06 mN^−1^ for the GC900 and GC700 SU-8 cantilevers, respectively. Since the fabricated device was originally designed for *GF* analysis of GC sensors, its sensitivity to surface stress is lower than the sensitivity to surface stress of other types of devices due to its relatively large thickness *h*^58,59^.

**Fig. 4:**
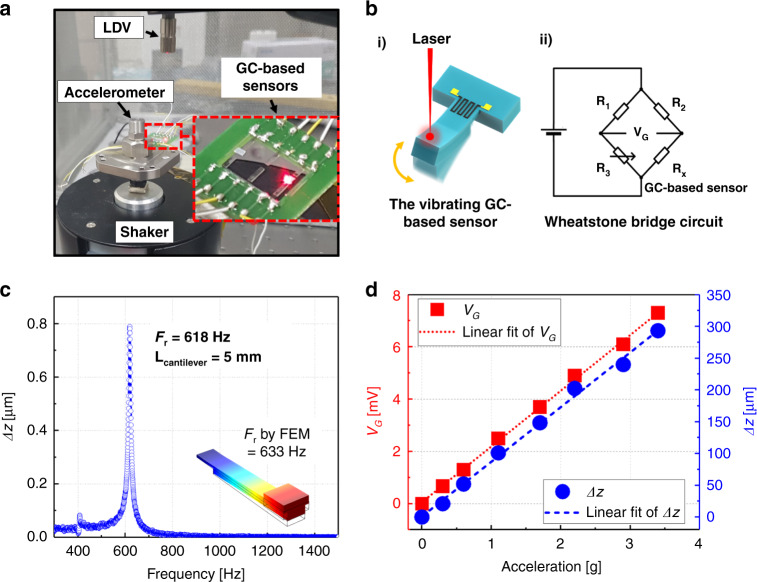
Vibrational characteristics of the glass-like carbon (GC)-based sensor. **a** Setup for measuring the dynamic response to acceleration of the GC-based sensor on the cantilever. The inset is a magnified image of the sample reflecting the laser beam focused at the end of the SU-8 cantilever. **b** (i) Schematic drawing of the vibrating GC-based sensor, and (ii) Wheatstone bridge circuit. **c** Measured mechanical displacement of the GC700-based cantilever for a periodic acceleration of frequency from 300 to 1400 Hz. The measured resonance frequency *F*_*r*_ is 618 Hz for the cantilever with a length of 5 mm. The inset shows a *F*_*r*_ of 633 Hz simulated by the finite element model (FEM). **d** Measured output voltage *V*_*G*_ and mechanical displacement as a function of the applied acceleration at the cantilever mechanical resonance frequency of 618 Hz

To date, GC has been used mainly in various MEMS applications, in particular as electrodes, but has not been applied as a mechanical sensor. Therefore, the purpose of this study is not simply to focus on the evaluation of piezoresistive properties but to demonstrate the possibility that our GC microstructures embedded in a polymer SU-8 cantilever can be used for mechanical sensors. For this reason, we investigated the dynamic behavior of GC sensors. A GC700-based cantilever with a proof mass to enhance the displacement sensitivity to acceleration was used^48^. The GC700 is mounted on an electromagnetic shaker. The mechanical displacement is measured using a laser Doppler vibrometer (LDV), as shown in Fig. 4a. Since the laser reflection is insufficient on the surface of the semitransparent SU-8, reflection tape (1 × 1 × 0.3 mm^3^) is attached to the end of the SU-8 cantilever (Fig. 4a). While the displacement of the GC-based SU-8 cantilever is being measured (Fig. 4b (i)), the Wheatstone bridge configuration (Fig. 4b (ii)) is used to sense the resistance variation of the GC strain sensor (*R*_x_) as a function of its dynamic vibration. The resistor values are *R*_*1*_ $\cong$ *R*_*3*_ $\cong$ 10 MΩ and *R*_*x*_ $\cong$ *R*_*2*_ $\cong$ 38 MΩ. A periodic chirp signal in the range of 100–5000 Hz producing an acceleration of 0.5 g is applied to the GC700-based SU-8 cantilevers by the electromagnetic shaker. Figure 4c shows the measured mechanical displacement of the SU-8 cantilever with a length of 5 mm. The measured resonance frequency is 618 Hz, slightly lower than the original resonance frequency without reflection tape, in agreement with FEA simulations (Fig. S9 of the Supplementary Information). As shown in Fig. 4d, the mechanical displacement and *V*_*G*_ are measured simultaneously while vibrating the SU-8 cantilever with an acceleration of 0–3.5 g at the resonance frequency of the cantilever (i.e*.*, 618 Hz). The measured displacement and the bridge differential voltage *V*_*G*_ increase linearly with the acceleration, with a sensitivity of 2.1 mV/g, demonstrating that the GC-based cantilever can be used as an accelerometer. The acceleration resolution, limited by the thermal noise of the GC piezoresistor, is ~300 μg/$\sqrt {{{{\mathrm{Hz}}}}}$. The resolution of the realized GC piezoresistive sensor is relatively poor compared to commercial MEMS accelerometers (~10 μg/$\sqrt {{{{\mathrm{Hz}}}}}$)^61^, but it can be improved by enhancing the *GF*^7,43^ of the GC piezoresistor and by the structural optimization of the device^48^. To indicatively assess their electromechanical robustness, the realized devices are excited in resonance with an acceleration of 2.2 g for more than 12 h. No measurable variations in electrical resistance, mechanical resonance frequency, or mechanical oscillation amplitude are observed.

## Conclusions and outlook

In this work, we investigated the piezoresistivity of GC thin films obtained by pyrolysis of epoxy-based negative photoresist SU-8. The measured gauge factors (*GF*s) are in the range between 2.0 and 3.5 depending on the pyrolysis temperature. These *GF*s are close to those of metals and similar to the *GF*s measured for nonoptimized a-C or single-layered graphene^4,19,20^. As suggested by the works of Dai et al*.*^43^, a possible approach to increase the *GF* of pure GC is to deposit nickel on the GC and anneal it at 850 °C, when glassy graphene is formed by crystallization. Another possible approach to improve *GF* is to combine GC with nanowires such as CNTs^7^. Alternatively, composites consisting of GC nanoplatelets with insulating polymers can be studied^7,16,62^. Interestingly, the *GF* of the GC700 sensor was ~1.8 times higher than the *GF* of the GC900 sensor, similar to what is observed in commercial bulk GC rods under tensile stress, where *GF* increases as *T*_*p*_ decreases from 600 to 1100 °C^41^.

According to the study by Hunt et al*.*, the intrinsic piezoresistivity of GC is largest at 600 °C, but in this study, GC is formed as a thin film. Therefore, the initial resistance value (*R*_*0*_) becomes excessively large, which lowers the resolution of the device (Δ*R*/*R*_0_). In addition, the resistance of the GC thin-film pyrolyzed at 600 °C is very high and exceeds the measurable range but can be reliably measured with the four-point probe system. Considering that the difference between the piezoresistivity of GC700 and GC900 is not large (~43%), we do not expect a significant difference between the GC devices pyrolyzed at temperatures between 700 and 900 °C.

Future studies should thus investigate the piezoresistive properties of GC films obtained at *T*_*p*_ above 1200 °C because the larger fullerene-like cluster of GCs may cause the onset of a significant intrinsic piezoresistivity, which has not been studied thus far^28^. Several piezoresistive materials, such as polymer-derived ceramics and silicon carbide, have been proposed for applications in harsh environments^63–65^. Considering the excellent properties and good compatibility with conventional MEMS processes of GC thin films, GC-based piezoresistive devices are well suited for special environments, such as implantable medical prostheses and chemical/heat-resistant devices. As mentioned above, the main purpose of this study was to investigate the piezoresistive characteristics of SU-8-derived GC pyrolyzed at various temperatures. The electrical properties of the fabricated GC thin films were characterized by measuring the electrical resistivity, carrier concentration, and mobility using the Hall effect. The structural characteristics of GC thin films as a function of the pyrolysis temperature were evaluated through Raman spectroscopy and TEM analysis, and the chemical composition of sp^2^ and sp^3^ contents of the GC was assessed via an XPS method. In addition, by utilizing C-MEMS technology, GC-based piezoresistive microresistors were realized on silicon wafers and transferred to SU-8 polymer cantilevers to create GC-based MEMS sensors. The sensitivities of the fabricated device with respect to strain, static force, its surface stress, and dynamic response were evaluated to demonstrate the suitability of GC as a base material for MEMS piezoresistors.

## Materials and methods

### Preparation of the SU-8-derived GC thin film

A 700 nm-thick wet-oxidized silicon dioxide (SiO_2_) insulating layer on the surface of a silicon wafer is used as the substrate. SU-8 (GM 1040, Gersteltec, Switzerland) is spin-coated on the SiO_2_ layer at 3616 rpm for 40 s to a thickness of 1 µm. The coated SU-8 was soft baked on a ventilated hotplate at 65 °C (5 min) and 95 °C (5 min) and exposed to a constant ultraviolet (UV) intensity of 49 mJ/cm^2^ by a mask aligner (MJB4, Süss Microtec SE, Germany). The wafer was postbaked at 65 °C (5 min) and 95 °C for 15 min. The wafer was diced into 15 × 15 mm^2^ chips to make the chips suitable for thin-film analysis. The pyrolysis process to transform SU-8 into GC was performed in a tube furnace (ATV Technologies GmbH PEO601, Germany) under a constant nitrogen gas flow of 2000 mL/min. Heating was applied in two steps. The first heating was from 25 to 200 °C with a 30-min holding time and then ramped to the target temperatures (600–900 °C) with a holding time of 1 h. The rate of temperature heating and cooling was 10 °C /min.

### GC thin film characterization

The sheet resistance of the GC thin films was measured using a four-point probe system (OmniMap RS75, KLA Tencor Corp., USA), as shown in the inset of Fig. 2a. The thickness of GC thin films was measured by a surface stylus profilometer (Dektak XT, Bruker Corp., USA). The resistivity is calculated as *ρ* = *R*_*s*_*t*, where *ρ* is the resistivity, *R*_*s*_ is the sheet resistance, and *t* is the thickness of the GC thin films. To investigate the details of the electrical conductivity of the GC, a Hall measurement system (8404; Lake Shore Cryotronics Inc., USA) was used to measure the carrier concentration (*N*), Hall mobility (*μ*_*H*_), and Hall coefficient (*R*_*H*_). We used samples with GC thin films formed on Si wafers with a size of 4 mm × 4 mm. The thickness of the GC thin film varies slightly depending on the *T*_*p*_, and the detailed thickness is listed in the Supplementary Information (Table S1). The measurements are conducted at room temperature in an alternating magnetic field with an amplitude of 1.2 T and a frequency of 0.1 Hz. The applied bias current is 0.5–1 mA.

The Raman spectra of the GC thin films were recorded by a confocal Raman spectrometer (Renishaw, UK) using a laser beam with an excitation wavelength of 532 nm. XPS was employed to investigate the surface of the GC thin films at room temperature in ultrahigh vacuum with a surface analysis system (ESCALAB 250Xi, Thermo Fisher Scientific Inc., USA). After etching with argon plasma for 30 sec, a beam with a diameter of 650 µm was applied to the GC thin films. The nanostructure of GC was observed by field emission transmission electron microscopy (TEM; HF-3300, Hitachi, Japan) at an accelerating voltage of 300 kV. The GC thin films were peeled off and placed onto a standard TEM grid.

### MEMS fabrication process of the SU-8-based GC piezoresistive sensor

Silicon wafers (p-type, 100 mm diameter, single-side polished) were used as substrates to fabricate the GC sensors. To initiate this process, a 700 nm-thick SiO_2_ layer is produced by wet oxidization and used as a sacrificial layer to release the device from the wafer at the final stage. A 1 µm-thick SU-8 (GM 1040, Gersteltec, Switzerland) is spin coated on the SiO_2_ layer to form the GC piezoresistor. The SU-8 layer is patterned by a negative photolithography process as follows: soft bake (5 min each at 65 and 95 °C), exposure dose (49 mJ/cm^2^), and postexposure bake (PEB; 5 min at 65 °C and 15 min at 95 °C). The SU-8 patterns were pyrolyzed in a tube furnace (ATV Technologies GmbH PEO601, Germany) under a constant N_2_ gas flow (2000 mL/min) with a two-step heating process consisting of a first ramp to 200 °C at 10 °C /min, followed by a holding time of 30 min and a second ramp up to the target temperature (i.e*.*, 700–900 °C) at 10 °C/min with a final holding time of 1 h. No delamination was observed between the GC and the substrate even after high-temperature pyrolysis at 900 °C. In a previous study^33^, the GC thin films were observed to be pyrolyzed at 1000 °C and can be successfully transferred to a polymer substrate. Fifty-nm-thick gold (Au) electrical pads were created by sputtering (Spider-600, Pfeiffer Vacuum GmbH, Germany) with a lift-off process by photolithography using AZ nLOF 2020 photoresist and AZ 726 MIF developer (MicroChemicals GmbH, Germany). The 100 µm-thick SU-8 (GM 1075, Gersteltec, Switzerland) structural layer is spin-coated, and after one hour of relaxation time for uniformity, soft baking is performed at low temperature (45 °C for 12 h) to minimize residual stresses that could cause cracking in the prepatterned GC resistors. UV exposure was performed at 350 mJ/cm^2^ by a mask aligner (MA6/BA6, Süss Microtec SE, Germany). PEB was also conducted at a low temperature of 45 °C for 12 h. The 200 µm-thick SU-8 is patterned as the device body structure by negative photolithography as follows: soft-bake (24 h at 45 °C), exposure (460 mJ/cm^2^), and PEB (24 h at 45 °C). Finally, the device was released from the Si wafer by etching the SiO_2_ sacrificial layer in a buffered oxide etchant (BOE 7:1, MicroChemicals GmbH, Germany) for 48 h. In a previous study^33^, it was also confirmed that the GC thin-film pyrolyzed at 1000 was successfully transferred to the polymer substrate.

### Measurement of the static response of the GC-based SU-8 cantilever

The mechanical bending force of the SU-8 cantilevers was measured by a microforce sensing probe (FT-S1000, FemtoTools AG, Switzerland) with a micromanipulator (FT-RS1002 Microrobotic System, FemtoTools AG, Switzerland). The SU-8 cantilever is attached on the positioning piezostage perpendicular to the sensing probe. The threshold force for the probe to find contact with the SU-8 cantilever is set to 150 µN, and the step for each measurement is 20 µN up to a maximum force of 8 mN to protect the mechanically fragile sensing probe. The resistance variation as a function of the movement of the sensing probe was measured with a digital multimeter (2701, Keithley, USA).

### Evaluation of the vibrational characteristics

The mechanical vibration is applied by an electromagnetic shaker (Logtech Corp., Korea) with a vibration controller system combined with an accelerometer (PV-41, RION Corp., Japan) for feedback control of the acceleration. The mechanical displacement was measured by a laser Doppler vibrometer (LDV; OFV-5000, Polytec GmbH, Germany). The Wheatstone bridge circuit connected to the device is placed in a Faraday cage to reduce ambient electrical noise. The output voltage of the Wheatstone bridge circuit is measured by the reference channel of the LDV.

## Supplementary information

Supplemental material
