# Supplementary material for: SU-8 cantilever with integrated pyrolyzed glass-like carbon piezoresistor
Source: Microsyst Nanoeng. 2022 Feb 10;8:22. doi: 10.1038/s41378-022-00351-9 (PMC8831616; doi:10.1038/s41378-022-00351-9)
Supplement: Supplementary file 1 — Supplemental material [file 41378_2022_351_MOESM1_ESM.docx]

**Supplementary Information**

**SU-8 cantilever with integrated pyrolyzed glass-like carbon piezoresistor**

**Jongmoon Jang,^1,2^ Giulia Panusa,^3^ Giovanni Boero,^1^ Juergen Brugger^1^***

*^1^Microsystems Laboratory, École Polytechnique Fédérale de Lausanne (EPFL), 1015 Lausanne, Switzerland*

*^2^Department of Functional Ceramics, Korea Institute of Materials Science (KIMS), 51508 Changwon, Republic of Korea*

*^3^Optics Laboratory, École Polytechnique Fédérale de Lausanne (EPFL), 1015 Lausanne, Switzerland*

*Corresponding Authors

*E-mail address*: juergen.brugger@epfl.ch (J. Brugger)

Tel: +41.21.693.6573;

**Table S1.** Sheet resistance, thickness, and resistivity of the SU-8 derived glass-like carbon thin films pyrolyzed at temperatures from 700 to 900 ˚C. The sheet resistance and thickness are the average value of five samples.

| Pyrolysis temperature [˚C] | 700 | 750 | 800 | 850 | 900 |
| --- | --- | --- | --- | --- | --- |
| Sheet resistance [kΩ/sq] | 55.7 ± 6.2 | 5.19 ± 0.90 | 1.69 ± 0.47 | 1.12 ± 0.21 | 0.897 ± 0.20 |
| Thickness [nm] | 144 ± 3.1 | 142 ± 6.3 | 133 ± 6.9 | 126 ± 2.4 | 123 ± 9.5 |
| Resistivity [10^-2^ Ωcm] | 80 ± 8.9 | 7.4 ± 1.2 | 2.3 ± 0.63 | 1.4 ± 0.27 | 1.1 ± 0.26 |

**Table S2.** Hall coefficient (*R_H_*), carrier concentration (*N*), and mobility ($\mu_{H}$) of the SU-8 derived glass-like carbon thin film pyrolyzed at temperatures from 700 to 900 ˚C. The reported values are the average of five samples.

| Pyrolysis Temperature [˚C] | 700 | 750 | 800 | 850 | 900 |
| --- | --- | --- | --- | --- | --- |
| Hall coefficient [cm^3^/C] | (2.4 ± 0.46) ×10^-1^ | (5.8 ± 3.0) ×10^-2^ | (9.2 ± 3.9) ×10^-3^ | (7.8 ± 1.6) ×10^-3^ | (5.2 ± 2.6) ×10^-3^ |
| Carrier concentration [cm^-3^] | (2.7 ± 0.59) ×10^19^ | (1.5 ± 1.0) ×10^20^ | (8.2 ± 4.6) ×10^20^ | (8.3 ± 1.8) ×10^20^ | (1.6 ± 1.0) ×10^21^ |
| Carrier mobility [cm^2^/V_s_] | 0.38 ± 0.30 | 0.41 ± 0.22 | 0.20 ± 0.075 | 0.32 ± 0.032 | 0.34 ± 0.20 |

**The dimension of the GC piezoresistors**

GC resistors are designed in a ‘meander shape'. The width of the conductor is 40 µm for all GC resistors. The length of the GC piezoresistive meander (*L_p_*) is covering 20% of the length of the cantilever (*L*)


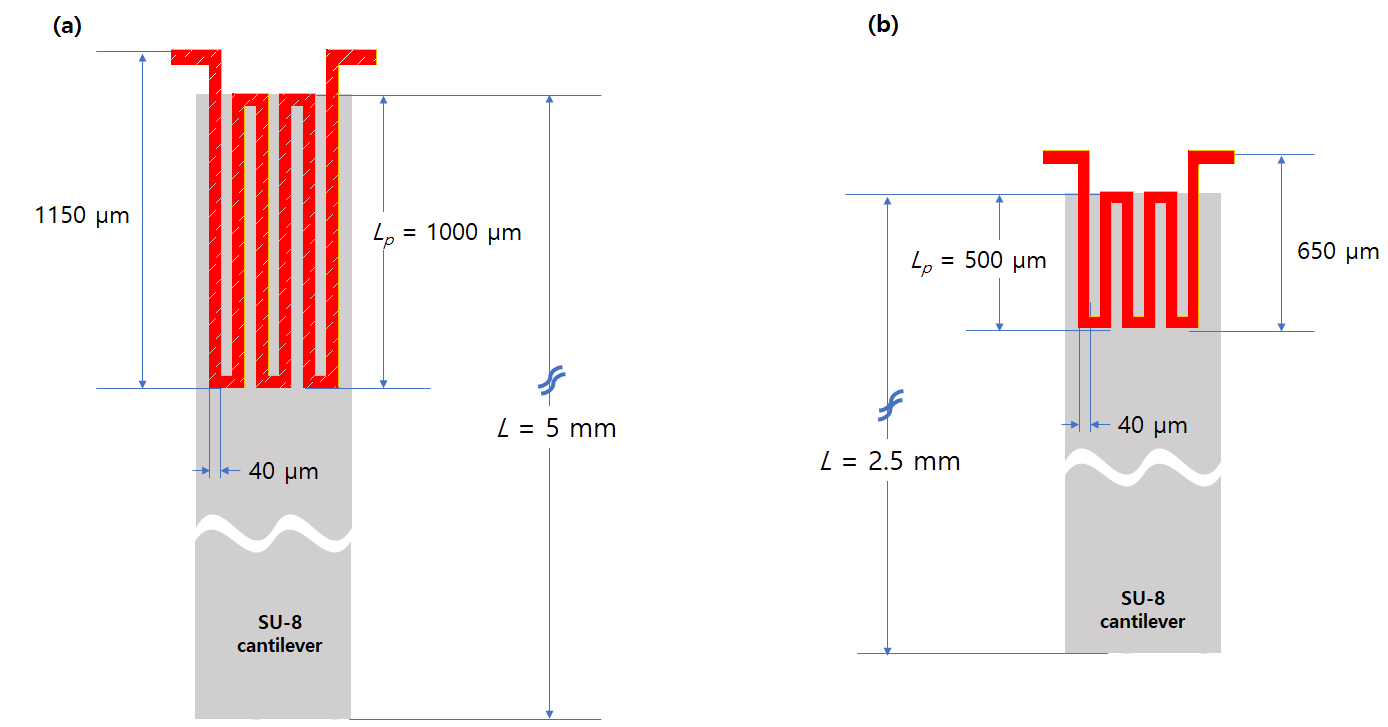


**Figure S1.** (a) The design of the GC resistor integrated with a 5.0 and (b) 2.5 mm SU-8 cantilever. In both cases, the length of the GC piezoresistive meander (*L_p_*) is 20% of the cantilever length (*L*; *i.e.,* 1000 µm for the 5 mm cantilever and 500 µm for the 2.5 mm cantilever). The width of the GC piezoresisitive meander is 440 µm for both.

**Raman spectroscopy for the GC thin films obtained at a pyrolysis temperature (*T_p_*) from 600 to 900 ^o^C.**

**
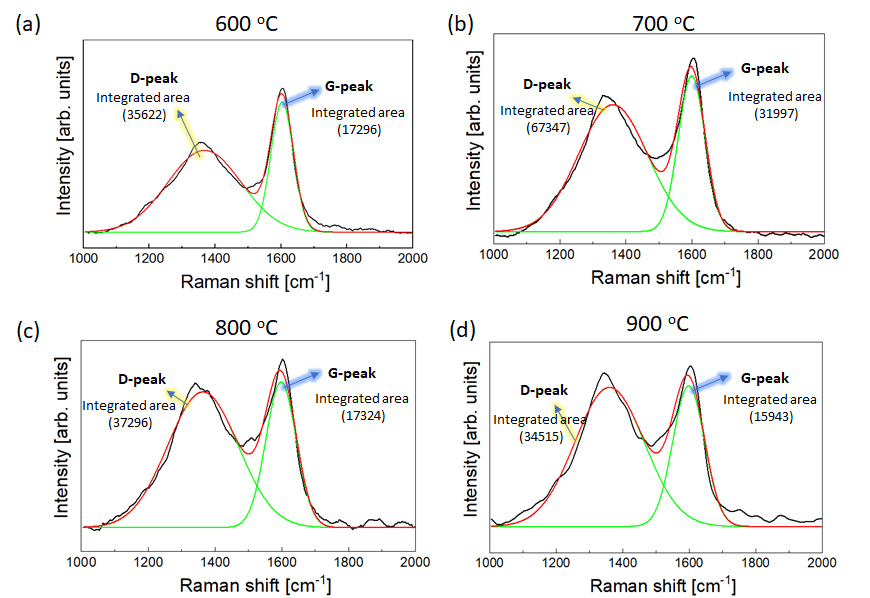
**

**Figure S2.** (a-d) Raman spectroscopy with the fitted spectra for the GC thin film obtained at a *T_p_* from 600 to 900 ^°^C

**X-ray photoelectron spectroscopy (XPS) C1s spectra for the GC thin films obtained at a pyrolysis temperature (*T_p_*) from 600 to 900 ^°^C.**


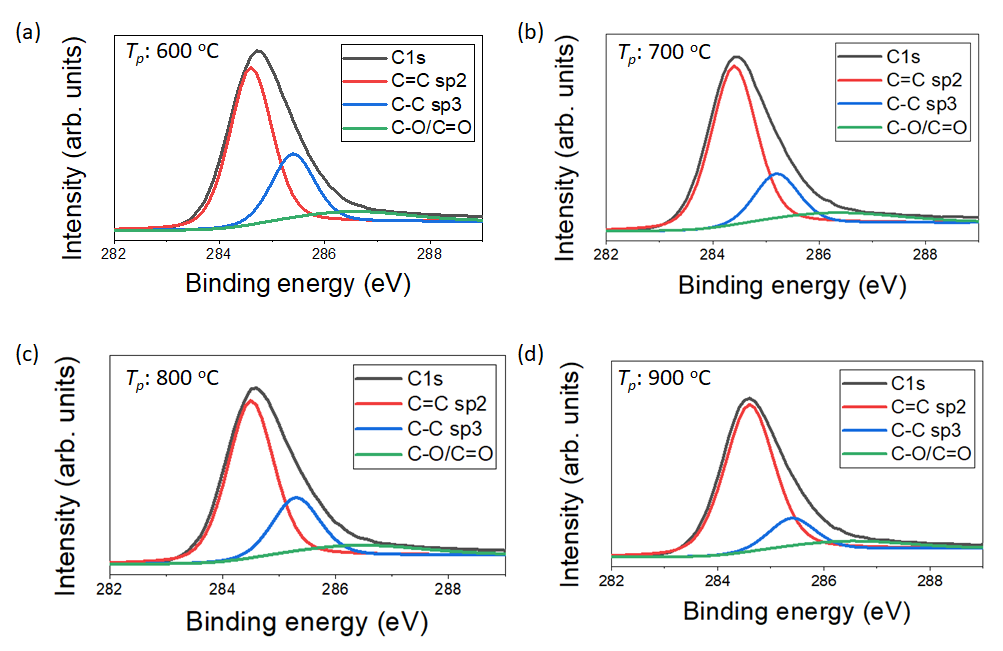


**Figure S3.** (a-d) XPS C1s spectra with the fitted spectra for the GC thin film obtained at a *T_p_* from 600 to 900 ^°^C

**Transmission electron microscopy (TEM) images of the SU-8 derived glass-like carbon**

**
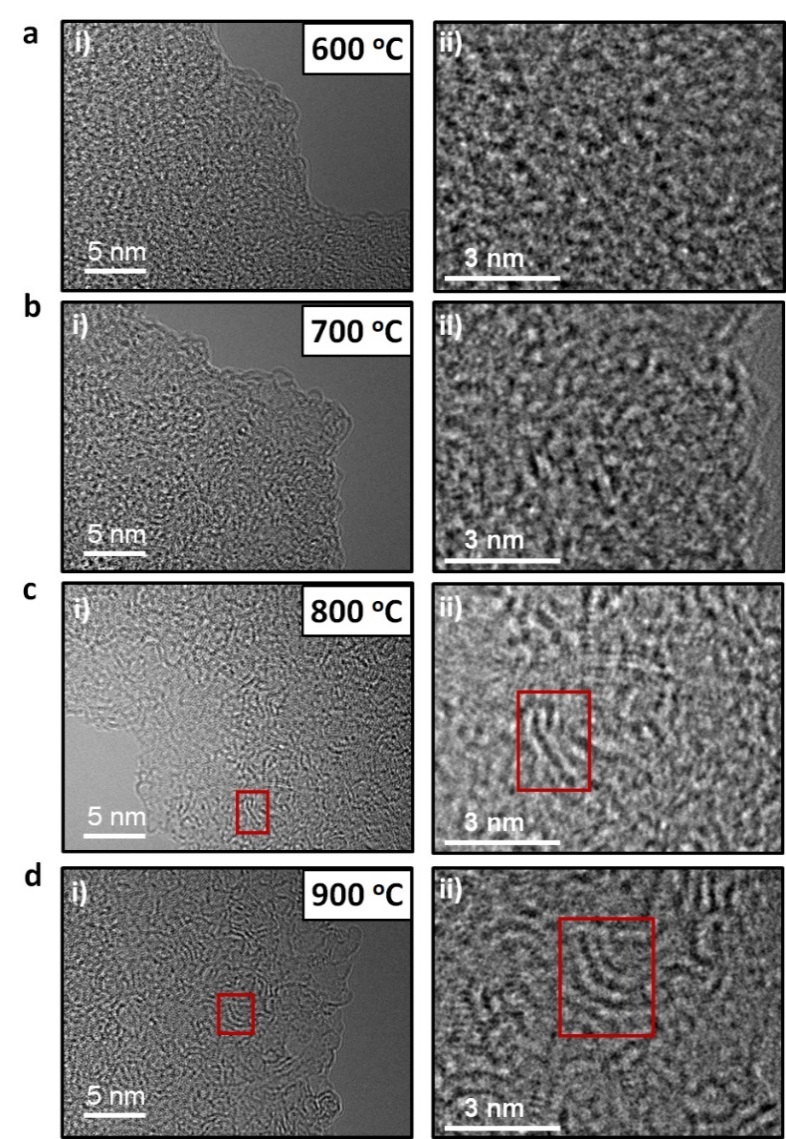
**

**Figure S4**. Transmission electron microscopy images of the GC thin film obtained by pyrolysis at temperatures of (a) 600, (b) 700, (c) 800, and (d) 900 ^°^C, respectively. The curled structures of the GC600 sample (*i.e.,* GC pyrolyzed a *T_p_* of 600 ^°^C) based on graphene-like domains are present, which consist of in-plane sp^2^ carbon and defects. As *T_p_* increased to 800 ^°^C, the curled layers became longer and have the tendency to form stacks (c). In the GC 900 sample, the layers are parallel, as shown in (d). The curvatures of the curled layers in GC800 and GC900 films correspond to the fragments of fullerene-like elements.

**Investigation of the surface morphology of the GC thin films by scanning electron microscopy (SEM)**

**
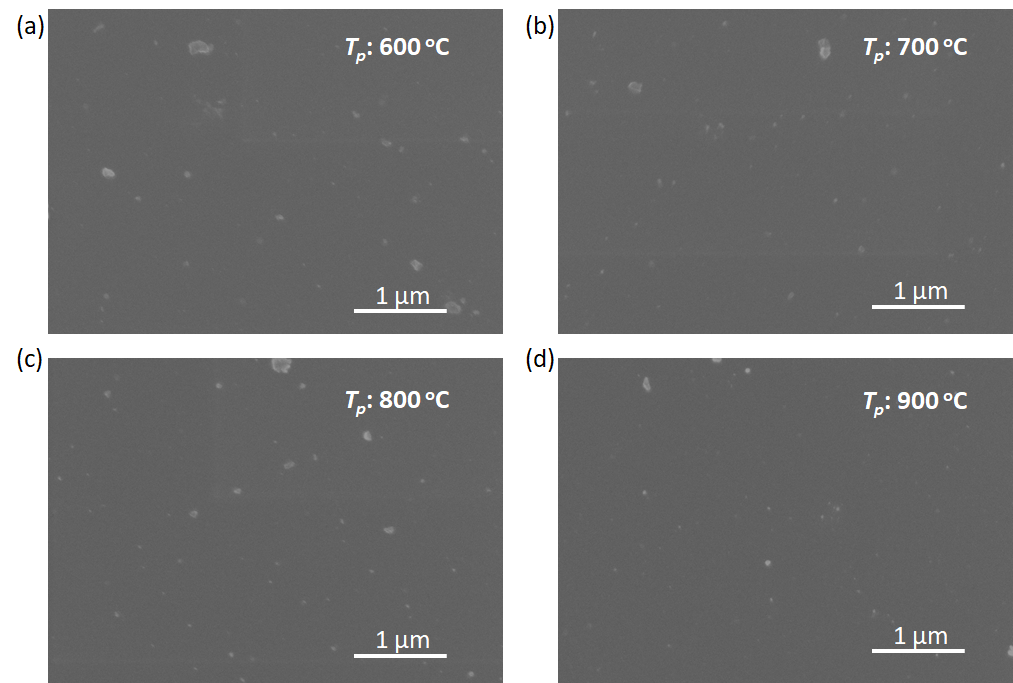
**

**Figure S5.** (a-d) Scanning electron microscopic (SEM) images of the GC thin-film pyrolyzed from 600 to 900 ^°^C. Each SEM image shows the surface morphology of SU-8 derived GCs formed after pyrolysis, and there are no significant differences among the samples pyrolyzed at different *T_p_*.

**Investigation of the surface morphology of the GC thin films by atomic force microscopy (AFM)**

**
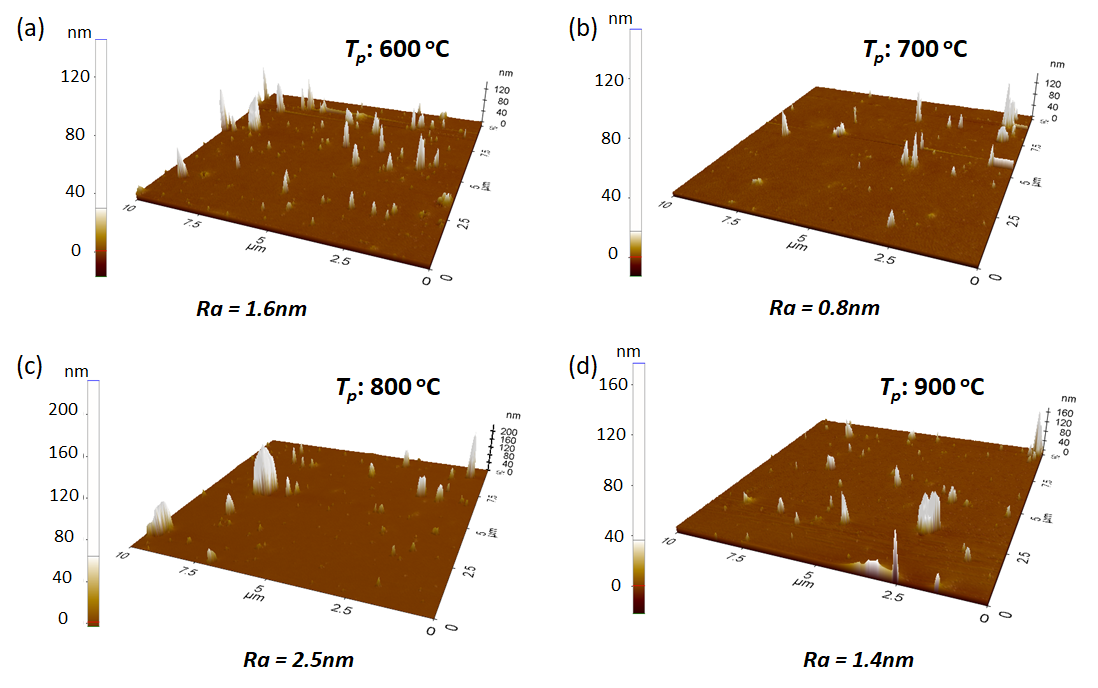
**

**Figure S6.** AFM images shows the morphological characteristic of the GC thin-film. The average roughness (*R_a_*) of the total area of each samples is (a) 1.6 nm at 600 ^°^C *T_p_*, 0.8 nm at 700 ^°^C *T_p_*, 2.5 nm at 800 ^°^C *T_p_*, and 1.4 nm at 900 ^°^C *T_p_*. No significant differences are observed for samples pyrolyzed at different *T_p_*_._

**Finite element analysis to simulate the spring constant of the GC900 and GC700 cantilevers in lengths from 2.5 to 5.0 mm**

When a mechanical force of 5 mN is applied to the tip end of the SU-8 cantilever of GC900 and GC700 with a length from 2.5 to 5.0 mm, the strain induced at the GC resistor and displacement at the tip end are analyzed by finite element analysis (COMSOL Multiphysics, USA).

**
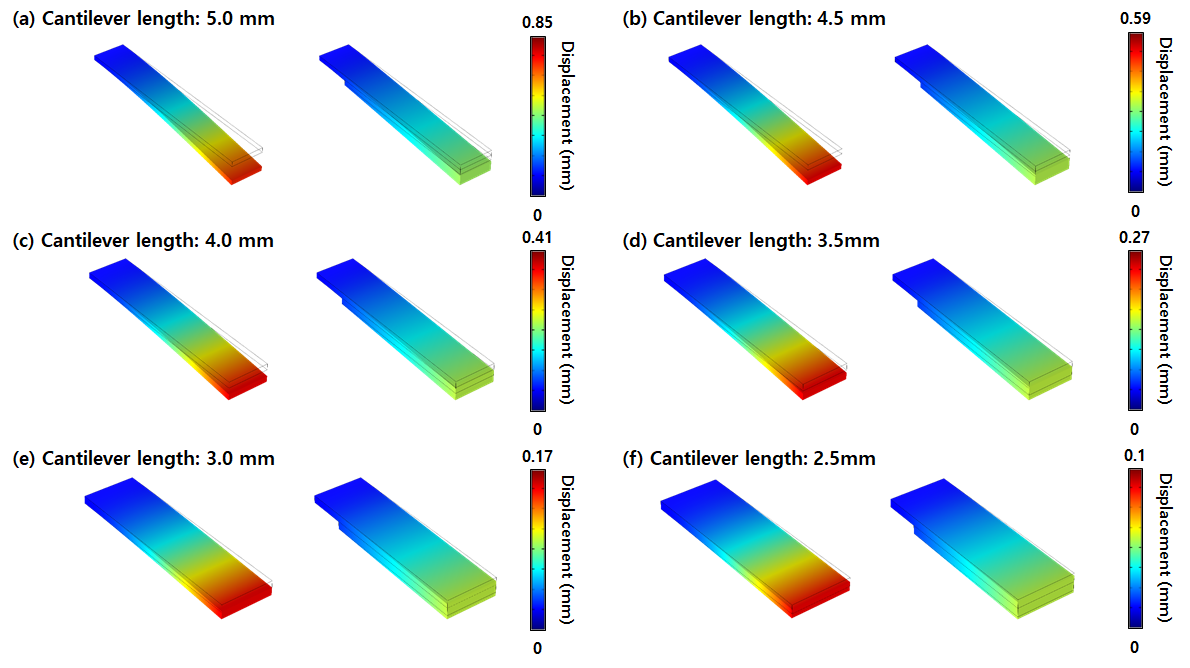
**

**Figure S7.** (a – f) Simulation of tip displacement of GC900 and GC700 with a length of 2.5 to 5 mm applying the same mechanical force at the end of the cantilever

**Theoretical simulation to compare the induced strain in the GC resistor for GC900 and GC700**

For the same tip displacement applied to the cantilever design with (GC700) and without proof mass (GC900), the average strain applied in the GC piezoresistor (*i.e.,* the range from anchor to 20 % of the total beam length) is simulated through finite element analysis. About 1.9 times higher mechanical strain than GC900 is applied to GC700.

**
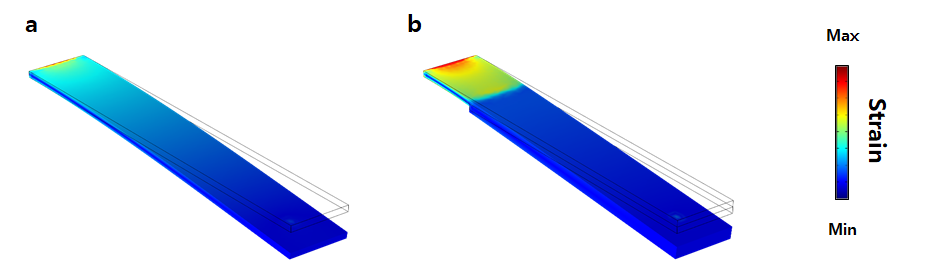
**

**Figure S8.** Simulation of applied strain of (a) GC900 and (b) GC700 SU-8 cantilever applying the same mechanical displacement at the end of each cantilever

**Finite element analysis of the resonance frequency of the GC-based strain sensor**

In the following, we report the results of the simulations of the resonance frequency (*F_r_*) of a GC700-based sensor with a length of 5 mm by finite element analysis (COMSOL Multiphysics, USA). Without reflection tape at the end of the SU-8 cantilever, (a) *F*_r_ is 868 Hz. With a reflection tape of 1 × 1 mm^2^, *F*_r_ decreases to 633 Hz. Increasing the size of the reflective tape to 1.3 × 1.3 and 1.5 × 1.5 mm^2^, *F*_r_ is 584 and 523 Hz, respectively.


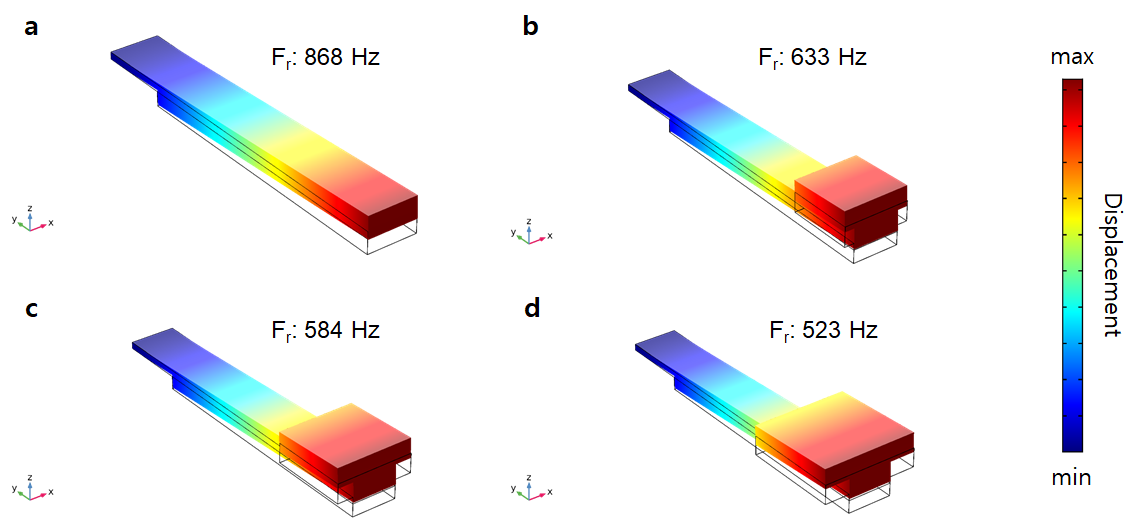


**Figure S9.** Simulation of the resonance frequency for the GC700-based sensor (a) w/o and (b) w/ a reflection tapes having a size of 1 × 1 mm^2^, (c) 1.3 × 1.3 mm^2^, (d) and 1.5 ×1.5 mm^2^.
